# Whole-exome sequencing in amyotrophic lateral sclerosis suggests *NEK1* is a risk gene in Chinese

**DOI:** 10.1186/s13073-017-0487-0

**Published:** 2017-11-17

**Authors:** Jacob Gratten, Qiongyi Zhao, Beben Benyamin, Fleur Garton, Ji He, Paul J. Leo, Marie Mangelsdorf, Lisa Anderson, Zong-Hong Zhang, Lu Chen, Xiang-Ding Chen, Katie Cremin, Hong-Weng Deng, Janette Edson, Ying-Ying Han, Jessica Harris, Anjali K. Henders, Zi-Bing Jin, Zhongshan Li, Yong Lin, Xiaolu Liu, Mhairi Marshall, Bryan J. Mowry, Shu Ran, David C. Reutens, Sharon Song, Li-Jun Tan, Lu Tang, Robyn H. Wallace, Lawrie Wheeler, Jinyu Wu, Jian Yang, Huji Xu, Peter M. Visscher, Perry F. Bartlett, Matthew A. Brown, Naomi R. Wray, Dongsheng Fan

**Affiliations:** 1 Queensland Brain Institute, The University of Queensland, Brisbane, QLD 4072 Australia; 2 Institute for Molecular Bioscience, The University of Queensland, Brisbane, QLD 4072 Australia; 3 Department of Neurology, Peking University Third Hospital, No 49, North Garden Road, Haidian District, Beijing 100191 China; 4 University of Queensland Diamantina Institute, The University of Queensland, Translational Research Institute, Brisbane, QLD 4102 Australia; 5 Institute of Health and Biomedical Innovation, Queensland University of Technology, Translational Research Institute, Brisbane, QLD 4102 Australia; 6 Laboratory of Molecular and Statistical Genetics and the Key Laboratory of Protein Chemistry and Developmental Biology of the Ministry of Education, College of Life Sciences, Hunan Normal University, Changsha, Hunan China; 7 Center for Bioinformatics and Genomics, Department of Global Biostatistics and Data Science, School of Public Health and Tropical Medicine, Tulane University, 1440 Canal St, Suite 2001, New Orleans, LA 70112 USA; 8 Center of System Biomedical Sciences, University of Shanghai for Science and Technology, 334, Jungong Road, Yangpu District, Shanghai 200093 China; 9 Division of Ophthalmic Genetics, Laboratory for Stem Cell and Retinal Regeneration, The Eye Hospital of Wenzhou Medical University, Wenzhou, 325027 China; 10 Institute of Genomic Medicine, Wenzhou Medical University, Wenzhou, 325027 China; 11 The Centre for Advanced Imaging, The University of Queensland, Brisbane, QLD 4072 Australia; 12 Department of Rheumatology and Immunology, Shanghai Changzheng Hospital, The Second Military Medical University, Shanghai, 200003 China; 13 Queensland Centre for Mental Health Research, The University of Queensland, Brisbane, QLD 4072 Australia

## Abstract

**Background:**

Amyotrophic lateral sclerosis (ALS) is a progressive neurological disease characterised by the degeneration of motor neurons, which are responsible for voluntary movement. There remains limited understanding of disease aetiology, with median survival of ALS of three years and no effective treatment. Identifying genes that contribute to ALS susceptibility is an important step towards understanding aetiology. The vast majority of published human genetic studies, including for ALS, have used samples of European ancestry. The importance of trans-ethnic studies in human genetic studies is widely recognised, yet a dearth of studies of non-European ancestries remains. Here, we report analyses of novel whole-exome sequencing (WES) data from Chinese ALS and control individuals.

**Methods:**

WES data were generated for 610 ALS cases and 460 controls drawn from Chinese populations. We assessed evidence for an excess of rare damaging mutations at the gene level and the gene set level, considering only singleton variants filtered to have allele frequency less than 5 × 10^–5^ in reference databases. To meta-analyse our results with a published study of European ancestry, we used a Cochran–Mantel–Haenszel test to compare gene-level variant counts in cases vs controls.

**Results:**

No gene passed the genome-wide significance threshold with ALS in Chinese samples alone. Combining rare variant counts in Chinese with those from the largest WES study of European ancestry resulted in three genes surpassing genome-wide significance: *TBK1* (*p* = 8.3 × 10^–12^), *SOD1* (*p* = 8.9 × 10^–9^) and *NEK1* (*p* = 1.1 × 10^–9^). In the Chinese data alone, *SOD1* and *NEK1* were nominally significantly associated with ALS (*p* = 0.04 and *p* = 7 × 10^–3^, respectively) and the case/control frequencies of rare coding variants in these genes were similar in Chinese and Europeans (*SOD1*: 1.5%/0.2% vs 0.9%/0.1%, *NEK1* 1.8%/0.4% vs 1.9%/0.8%). This was also true for *TBK1* (1.2%/0.2% vs 1.4%/0.4%), but the association with ALS in Chinese was not significant (*p* = 0.14).

**Conclusions:**

While *SOD1* is already recognised as an ALS-associated gene in Chinese, we provide novel evidence for association of *NEK1* with ALS in Chinese, reporting variants in these genes not previously found in Europeans.

**Electronic supplementary material:**

The online version of this article (doi:10.1186/s13073-017-0487-0) contains supplementary material, which is available to authorized users.

## Background

Amyotrophic lateral sclerosis (ALS) is a progressing motor neuron disease characterised by loss of function (LOF) of motor neurons, which are essential for controlling voluntary muscle activity such as walking, breathing and speaking. This condition leads to premature death with a median survival of about two to three years. Disease likely arises from a combination of genetic susceptibility [1–3] and environmental factors [4]. However, our understanding of what these factors are and how they contribute to disease risk, onset and progression remain incomplete.

Likely due to this limited understanding of disease aetiology, there has been limited success in designing any effective treatment for ALS. To date, the most important fundamental insights into the underlying cellular mechanisms have resulted from genetic studies of the known causal mutations [5]. However, highly penetrant identified mutations still only account for up to 10% of cases [6, 7] and thus more work needs to be done. Identification of both causal and risk genes will help build a more complete picture of the underlying mechanisms and pathways for disease and any new ALS molecule is potentially a new therapeutic target [8].

Whole-exome sequencing (WES) studies designed to identify genes enriched for rare variants have been conducted for ALS. Association testing has typically been conducted at the gene level comparing the burden of rare coding variants in cases vs controls. Large sample sizes are needed to detect significant associations due to testing of ~ 20,000 genes and because the multiple testing burden is often increased by considering different genetic models. The largest study to date, comprising 2874 cases and 6405 controls of European ancestry, identified the known ALS gene *SOD1* as the only gene passing the multiple-testing corrected threshold for significance of association [9]. A follow-up study of 51 genes in an independent sample of 1318 cases and 2371 controls identified *TBK1* as a novel ALS risk gene [9] (discovery association *p* = 1.13 × 10^−5^, replication *p* = 5.78 × 10^−7^ and combined *p* = 3.63 × 10^−11^), with later GWAS support for association of common single nucleotide polymorphisms (SNPs) in the same locus (*p* = 6.6 × 10^–8^) [10]. A second gene, *NEK1*, was highlighted as suggestively significant. Both *TBK1* and *NEK1* are notable because protein–protein interaction analyses link them with other known ALS genes.

The next largest WES study of ALS, a case-control (1022 cases vs 7315 controls) study with cases selected as index individuals from families with multiple recorded cases of ALS (fALS) [11], identified *NEK1* as the only significant gene after correcting for multiple testing (ten known ALS genes had been excluded from the analysis to train modelling parameters). Follow-up analysis in four ALS cases from an isolated Dutch community suggested p.Arg261His as a specific *NEK1* candidate variant. An association analysis for this variant in 1022 familial ALS (fALS) plus 6172 sporadic ALS (sALS) cases compared to 11,732 controls found the allele frequency at this locus to be 0.81% in cases compared to 0.35% in controls (odds ratio [OR] = 1.41, *p* = 1.2 × 10^–7^), thus confirming *NEK1* as an ALS risk gene.

The vast majority of published human genome-wide studies, including for ALS, have used samples of European ancestry. The importance of trans-ethnic studies in human genetic studies is widely recognised [12–14], yet a dearth of studies of non-European ancestry remains. In Asians, the lifetime risk of ALS is estimated to be lower (0.1%) [15] than in Europeans (0.3%) [16] and the mean age of onset is estimated to be a few years earlier [17, 18]. This may reflect the different frequencies of many gene variants, including those already identified as risk or causal [19]. For example, *SOD1* mutations account for a higher proportion of Asian familial cases compared to European familial cases (30 vs 14.8%) [20], while the reverse is true for the *C9orf72* repeat expansion in sALS cases (~5% in Europeans [20] compared to only 0.3% [21] in Asians), likely due to different founder events, and with evidence that it may have arisen on a different haplotype background [21]. Here, we report the largest WES study for ALS in Chinese to date.

## Methods

### Participants

The samples are a subset of previously published genome-wide association study (GWAS) data of 1324 cases and 3115 controls [22], which were selected for WES based on DNA availability (627 cases and 186 controls). All cases and controls are of Chinese origin from Mainland China. Additional Chinese ancestry controls were provided through collaboration with the Hunan Normal University and the University of Shanghai for Science and Technology (HNU; 86 individuals) and Wenzhou Medical University (WMU; 479 individuals) (Additional file 1: Table S1). The WMU controls are individuals who attended the affiliated hospitals of Wenzhou Medical University with no medical or family history of neurological disorders during the years 2007–2015.

### Whole-exome sequencing data

WES data were generated on 611 Chinese sporadic ALS cases (including two *C9orf72* carriers), 16 familial cases (those with one or more affected first-degree relatives) and 186 controls. Only the cases were screened for *C9orf72* repeat expansion. Samples were indexed and multiplexed in groups of six per lane and sequenced in 101-bp paired-end mode using the Illumina HiSeq 2000 platform, but with a range of capture kits (see Additional file 1: Table S1 for full details). Of note was that HNU samples (*n* = 86) differed from the other samples in terms of capture kit (NimbleGen SeqCap EZ Exome v2) and in mean on-target coverage (~ 18.0X overall and 13.8X in v3 capture regions compared to ~ 40–50X for other samples).

Since rare variants are less likely to be called if coverage is low, and if differences in coverage are confounded with affected status, as is the case with our HNU controls, then analyses involving case-control comparisons may be biased. To minimise the potential for this problem, we created two sets of samples: one excluded HNU controls (610 cases and 460 controls after quality control [QC]) and the other included HNU controls (610 cases and 545 controls after QC) but was restricted to variants common to the NimbleGen v2 and v3 capture kits (*n* = 187,512 post-QC SNPs, compared to 446,395 post-QC SNPs for the primary analysis excluding HNU controls; see below for variant calling criteria). QC and analysis of the two sets of samples was performed separately but using the same analytical pipeline. The results of analyses excluding (presented in the main text) and including (Additional file 1: Table S2) HNU controls do not impact the conclusions drawn.

### Variant calling

Image processing and sequence extraction were performed using the standard Illumina Genome Analyzer software. The samples were de-multiplexed using CASAVA (v1.8.2) outputting the short reads for each individual sample in ‘fastq’ format. The quality of all raw sequencing reads (also including WMU and HNU controls) was evaluated using the FastQC (v0.10.1) software. We generated ~ 5.94 Tbp of sequence data for a total number of 813 individuals (611 sporadic cases, 16 familial cases and 186 controls), with a mean on-target coverage of 42.42X per individual. In addition, we analysed ~ 3.18 Tbp of sequence data (mean on-target coverage of 45.01X per individual) for 479 WMU controls and ~ 0.16 Tbp of sequence data (mean on-target coverage of 13.83X per individual) for 86 HNU controls.

Sequence alignment and variant calling were performed using the same BWA-Picard-GATK analysis pipeline for all 1378 samples. Briefly, we aligned the paired-end reads to the human reference genome (hg19) using BWA (v0.6.2) [23], performed file conversion from SAM to BAM and generated the sorted and indexed BAM files using SAMtools (v0.1.17) [24], and marked duplicates using the Picard software package (http://broadinstitute.github.io/picard/) (v1.72). We then used GATK (v3.4-0) [25] to perform ‘Indel Realignment’, ‘Base Quality Score Recalibration’, ‘Variant Calling’ (GATK HaplotypeCaller in a gVCF mode), ‘Joint Genotyping’ and ‘Variant Recalibration’ as described in the GATK Best Practices [26] guidelines. Variants tagged as ‘PASS’ by the GATK Variant Quality Score Recalibration (VQSR) module were used for downstream analysis. The GATK resource bundle (v2.5) was used for VQSR, which includes as training data known SNP sites from HapMap v3.3, the Illumina Omni2.5 array, the 1000 Genomes Project phase 1, dbSNP v137, and the Mills [27] and 1000G gold standard indels. The VQSR target sensitivity cut-offs were set to 99.5% for SNPs and 99% for indels. Variants in each individual were required to have a genotype quality score (GQ) of ≥ 20 for further analysis. The analysis-ready variants from the GATK analysis pipeline were annotated using the ANNOVAR software tool (version 2015 June 17) [28].

### Quality control

After the variants were called and annotated, we performed QC steps on individuals and variants (Additional file 1: Table S3). Briefly, individual-level QC was based on common SNPs (MAF > 1%) with genotype call rate > 95%. We excluded individuals from the association analysis who: (1) were sex-discordant/ambiguous (20 individuals); (2) had a genotyping call rate < 80% (123 individuals); (3) had an excessive heterozygosity rate (> 3 standard deviations from the mean; 52 individuals; (4) were shown to be ancestry outliers based on the first two principal components (PCs) derived from common SNPs (i.e. > 6 SD from Chinese CHB mean; 34 individuals); and (5) had a genetic relationship matrix value of > 0.1 with another individual (107 individuals from the WMU sample, known relatives). After QC, we had in total 1070 individuals (610 cases and 460 controls; 626 men and 444 women) remaining for the analyses. We performed the same QC steps for the common capture set. The total number of individuals after QC was 1155 (610 cases and 545 controls). After obtaining clean sets of individuals, we excluded genetic variants based on the following criteria: (1) low genotype call rate < 99%; (2) deviation from Hardy–Weinberg Equilibrium in controls (*p* < 10^–6^); (3) differential missingness between cases and controls (*p* < 10^–6^); and (4) ≥ 3 alleles.

### Gene-based burden analysis

We assessed evidence for an excess of rare damaging mutations in ALS cases compared to controls at the gene level using the SKAT-O test [29] implemented in the R SKAT package [30]. We used the SKAT-O test because it optimally combines the burden test, which is most powerful when a high proportion of variants in a gene are causal and have the same direction of effect, with the sequence kernel association test (SKAT), which is best used when only a small proportion of variants in a gene are causal or if both risk and protective variants are present. In order to facilitate meta-analyses of our results with Cirulli et al. [9], we followed their approach for variant filtering and classification of three variant sets under a dominant genetic model. Briefly, we analysed RefSeq genes for each of three variant sets: (1) all non-synonymous variants (‘Dominant coding’); (2) non-synonymous variants excluding those predicted to be benign by PolyPhen-2 [31] (‘Dominant not benign’); and (3) LOF variants, including stop-loss, stop-gain and splicing variants but not frameshift indels due to recognised difficulties calling indels from WES data [32] (‘Dominant LOF’). For consistency with Cirulli et al., we restricted our analyses to variants passing an internal frequency filter of < 5 × 10^–4^ (corresponding to singleton variants in our sample) and additionally applied a frequency threshold of < 5 × 10^–5^ in ExAC [33]. RefSeq genes with at least one qualifying variant were analysed for a total of 301,368 tests and a Bonferroni corrected *p* value of 1.66 × 10^–7^. SKAT-O tests were corrected for sex and the top ten PCs based on HapMap3 SNPs. We used default settings in the R SKAT package, including for imputation of missing genotypes and re-sampling methods for computing *p* values.

### Gene-set analyses

We performed gene-set burden testing in ALS cases compared to controls, as one means of overcoming study power limitations due to sample size. Briefly, we defined three curated gene-sets: (1) 30 genes robustly associated with risk of ALS; (2) 128 genes associated with risk of ALS (comprising 21 ALS risk genes, 77 ALS candidate genes and the 30 high confidence ALS genes in set 1); and (3) 245 genes associated with risk of ALS (128 genes in set 2) and/or any of five related neuromuscular disorders (fronto-temporal dementia, Charcot–Marie–Tooth disease, hereditary spastic paraplegia, hereditary ataxia, distal myopathy; total of 117 genes) (Additional file 1: Table S4). Qualifying variants were defined as above, for a total of nine gene-set tests (Bonferroni corrected *p* value for significance = 5.56 × 10^–3^) (Additional file 1: Table S2). The mean coverage of exonic regions for each gene was 29.16X with individual gene coverage (including 43 that were covered < 10X in cases or controls) provided in Additional file 1: Table S4.

### Meta-analysis of European and Chinese variant counts

We used a Cochran–Mantel–Haenszel test to evaluate evidence for association at the gene level in a combined analysis of case-control variant counts in Europeans [9] and our Chinese WES cohort. Each variant set count was separately analysed as described above for gene-based burden testing within our Chinese cohort. Considering genes with at least one qualifying variant in either cohort, we performed a total of 26,214 tests across the three variant classes (Bonferroni corrected *p* value threshold of 1.91 × 10^–6^) and we used the Breslow–Day test to assess evidence for homogeneity of ORs for each gene across Chinese and European samples [9].

### ALS-variant analysis

To identify known variants previously associated with ALS, cases and controls were screened for any of 1158 ALS variants previously reported in the Human Gene Mutation Database (HGMD, trial professional version, accessed 3rd May 2016) and Amyotrophic Lateral Sclerosis online Database (ALSoD, accessed 1st September 2016) [34] using ANNOVAR [28]. Since variants in these databases may include false positives (benign) or risk variants (i.e. they occur at a population frequency that is inconsistent with the assumed disease prevalence and penetrance), we ignored any known variants identified in our cohort for which the frequency in ExAC populations of any ethnicity (the ‘popmax’ approach [33]) was > 0.01. To identify novel variants in relevant genes we used a previously curated hierarchical gene-set [35] (Additional file 1: Table S4) and restricted the analysis to non-synonymous (missense), stop-gain/loss (nonsense) and splicing (first and last two bases of each intron) variants. To enhance pathogenicity call rates [36], any missense variants classified as ‘tolerated’ by both MetaLR [37] and MetaSVM_pred [37] (integration of 18 current deleteriousness-scoring methods) were excluded. ExAC [33] popmax MAF filters of < 5 × 10^–5^ and < 0.01 for dominant and recessive genetic architectures, respectively, were applied. These filters for novel variants in known disease genes were more stringent than the filters applied for gene-based testing (described above and adopted from Cirulli et al. to enable meta-analysis of gene-based variant counts) because the objective was to screen for putatively pathogenic variants. Final variant lists were cross-checked with clinical databases (OMIM, Clinvar [38]) and the literature for case reports to assess pathogenicity. In examining the curated set of genes [35] (Additional file 1: Table S4), variants passing all filters present in ≥ 1 individual (case and/or control) were identified.

Putatively pathogenic indels were screened for in a subset of 21 genes, with prior evidence for causative indels and/or LOF variants [35] (Additional file 1: Table S4). These were separated into non-truncating (in-frame) and truncating (frame-shift) insertions and deletions, which were subsequently cross-checked for pathogenicity as above.

## Results

**Fig. 1:**
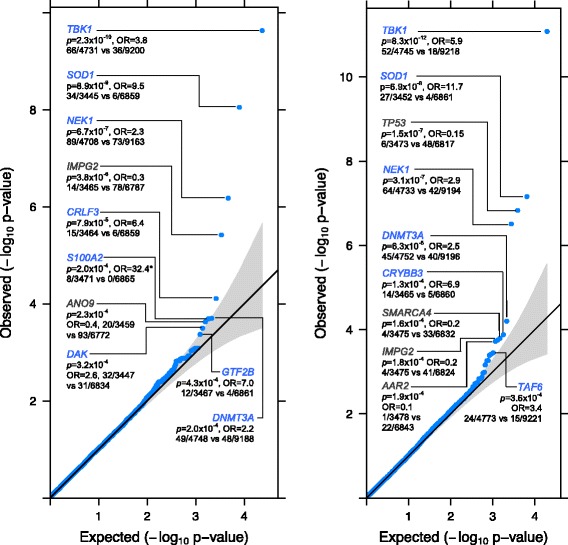
*Quantile–quantile plots* of the analysis of rare variant counts in combined Chinese and European data (up to 4797 cases and 9236 controls). The Cochran–Mantel–Haenszel test was applied to qualifying variants under three models: (L) dominant coding; (R) dominant not benign; and dominant LOF (Additional file 2: Figure S1). Test statistics are provided for the genes with the top ten associations (*blue* = increased risk, *grey* = reduced risk; *no qualifying variants were observed in controls for gene *S100A2*, so the OR was estimated by adding 0.5 to each cell of the largest cohort). The Bonferroni-corrected significance threshold was *p* ≤ 1.9 × 10^–6^, based on 26,214 tests across 18,117 genes. The genomic inflation factor, lambda (λ), was 1.069 for the dominant coding analysis and 1.067 for the dominant not benign analysisrecognised in our Chinese sample

In exome-wide gene-based association testing, no single gene was significantly associated with ALS after multiple testing correction (Additional file 1: Table S5, Additional file 2: Figure S1). This is unsurprising given the size of the sample. Similar to Cirulli et al. [9], we found that many of the top ranked genes, based on burden tests, showed an excess of rare mutations in controls compared to cases. Despite joint calling of variants, this likely reflects ascertainment associated with the additional control samples to increase our control sample size. When we meta-analysed per-gene case-control counts of rare functional mutations in our Chinese sample with those from the largest WES study of European ancestry [9] (Additional file 1: Table S6), three genes surpassed genome-wide significance for association with ALS with smaller *p* values than in the European ancestry samples alone: *TBK1*; *NEK1*;and *SOD1* (Table 1; Fig. 1). Both *NEK1* and *SOD1* were nominally significant in our Chinese sample, while *TBK1* was not significant (Table 1), and the case-control frequencies of rare coding variants were similar to Europeans (*NEK1* 1.8%/0.4% vs 1.9%/0.8%; *SOD1*: 1.5%/0.2% vs 0.9%/0.1%; *TBK1*: 1.2%/0.2% vs 1.4%/0.4%). We found no evidence for an excess of rare coding variants in cases in any of three a priori sets of genes associated with risk of ALS or related neuromuscular disorders (Additional file 1: Table S4).

**Table 1:** Genes identified from analysis of rare variant counts in combined Chinese and European ancestry data

Gene	Model	European *p* value^a^	Chinese *p* value^b^ (Case/control)^c^	Combined *p* value^d^	Combined OR (low/high)
*NEK1*	Dom coding	4.7 × 10^–6^	6.7 × 10^–3^ (11/2)	6.6 × 10^–7^	2.3 (1.6/3.2)
	Dom not benign	2.2 × 10^–6^	5.0 × 10^–2^ (7/0)	3.1 × 10^–7^	2.9 (1.9/4.4)
	Dom LoF	3.2 × 10^–9^	3.8 × 10^–1^ (2/0)	1.1 × 10^–9^	8.2 (3.7/20.7)
*SOD1*	Dom coding	7.1 × 10^–8^	3.7 × 10^–2^ (9/1)	8.9 × 10^–9^	9.5 (3.8/28.4)
	Dom not benign	3.9 × 10^–7^	5.3 × 10^–2^ (8/1)	6.9 × 10^–8^	11.7 (3.9/47.5)
	Dom LoF	NA	NA	NA	NA
*TBK1*	Dom coding	1.3 × 10^–9^	1.4 × 10^–1^ (7/1)	2.3 × 10^–10^	3.8 (2.4/5.9)
	Dom not benign	3.6 × 10^–11^	1.9 × 10^–1^ (6/1)	8.3 × 10^–12^	5.9 (3.3/10.8)
	Dom LoF	1.6 × 10^–6^	2.5 × 10^–1^ (1/0)	9.6 × 10^–7^	13.1 (3.7/70.9)

^a^Cochran–Mantel–Haenszel test (Cirulli et al., 2015) [9]

^b^SKAT-O test [29]

^c^Number of Chinese case carriers and control carriers out of 610 cases and 460 controls

^d^Cochran–Mantel–Haenszel test

**Fig. 2:**
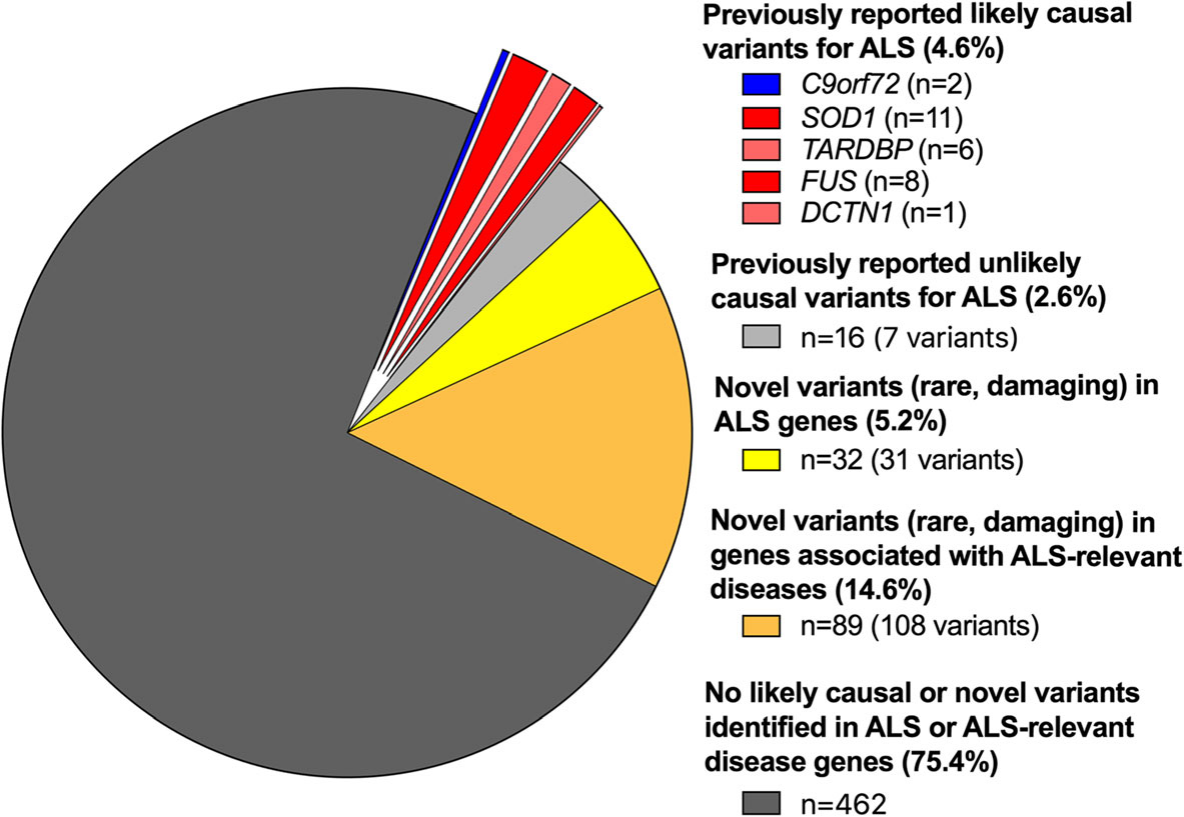
Summary of rare variants in Chinese WES sample comprising 597 sporadic (sALS) and 13 familial (fALS) cases. The screening of WES data of Chinese ALS cases identified ~ 5% with previously reported likely causal variants. Variants previously reported for ALS but now found to have population frequency (0.00005 ≤ freq < 0.01) are classified as ‘unlikely causal’. For variants identified in cases only, a number of putatively damaging, rare (MAF < 0.00005 dominant or < 0.01 recessive) variants in a predefined set of known ALS-priority genes (*n* = 32 cases) and ALS-relevant genes (*n* = 89 cases) were identified, but these have uncertain significance. Considering only fALS probands (*n* = 13), WES identified previously reported likely causal variants in five cases (1 *DCTN1*, 2 *FUS*, 1 *SOD1*, 1 *TARDBP*) with uncertain significance variants (damaging rare in ALS-relevant genes) in four others. Four percent of cases (24/610) and 3% of controls (13/460) were identified to be carrying one or more rare variants in ALS genes (from any category; causal, risk, candidate) and/or similar disease genes (Additional file 1: Table S10), but no individual harboured more than one likely causal variant. The number of cases are defined in the legend and expressed a percentage of total ALS case exomes screened (*n* = 610)

It is well-recognised that many variants reported in databases as ‘pathogenic’ for disease occur at a population frequency too high to be consistent with the reported disease prevalence [33, 39]. With this in mind, WES variants were screened for previously reported ALS variants for which we judged the evidence for pathogenicity was strong. Twenty-one of the Chinese sALS cases, five fALS probands and two of the controls harboured such variants (Additional file 1: Table S7; see Additional file 1: Table S8 for details of variants in *NEK1*, *SOD1* and *TBK1* that passed filters for gene-based testing, screening of known ALS variants or both). Considering exome variant results and two *C9orf72* carriers jointly, likely pathogenic variants account for 4.6% of ALS cases (28 out of 610) and 0.4% of controls (two out of 460; Fig. 2). This was slightly lower than the proportion of ALS cases with a known causal variant in an Australian clinical ALS cohort (~ 90% European ancestry) which was 10% using an identical filtering technique [35]. For familial probands, 38% (5 out of 13) were carriers of a likely causal variant. This is on the lower end of the range (30–70%) compared to what has previously been reported in European ancestry populations [35, 40]. The lower proportion of identified likely causal variants in both sALS and fALS cases is likely to be explained by a lower prevalence of the *C9orf72* repeat expansion which accounts for up to 7% of sALS and 40% of fALS in European populations [2] compared to just 0.3% in sALS cases in this study (as found in other Chinese samples [41, 42]). In contrast, we found a relatively high number of *NEK1* variants (nine non-synonymous variants in ten cases) and notably this did not include the recently reported p.Arg261His *NEK1* variant identified in a Dutch study [11]. While this can be expected given that ultra-rare variants tend to be highly population-specific [33], it is interesting that this locus has been independently.

## Discussion

In the largest WES study of ALS in Chinese samples we did not identify any specific gene significantly associated with ALS. Meta-analysing Chinese and European WES data strengthened the evidence for three genes (*SOD1*, *NEK1* and *TBK1*) reported as significantly associated with ALS in European samples (Table 1, Additional file 1: Table S6). The estimated case-control frequencies of rare coding variants in these genes in Chinese was similar to that reported for Europeans, and thus the nominal statistical associations we report for Chinese (Table 1) are a reflection of the available sample size. While *SOD1* is recognised as the most important ALS-associated gene in Chinese [20], evidence that *NEK1*, recently identified in European samples, may also be associated with ALS in Chinese is novel. Larger Chinese samples with whole exome data will be needed to confirm this result and to establish if *TBK1* is also an ALS gene in Chinese. Given the possible differences in genomic architecture of ALS between populations, additional genomic studies of ALS in non-European populations are warranted.

Assessing novel variants in known ALS disease genes revealed > 30 distinct mutations in *SOD1*, *TARDBP*, *CHMP2B*, *ERBB4*, *DCTN1*, *FIG4*, *FUS*, *MATR3*, *NEK1*, *SETX*, *SQSTM1*, *TBK1* and *UBQLN2* that were present in cases but not controls (Additional file 1: Table S9). Characterising the function of these newly identified variants, with respect to other reported variants and disease penetrance, is expected to enhance the ability to understand exactly how gene function and any related genes and/or pathways are impacted to alter ALS risk. Given the size of our cohort, we expect the variants identified to be typical of other clinical cohorts in China (Fig. 2), which will help to provide an evidence-based approach to the design of a targeted genetic screen, and may in future contribute to improved treatment strategies. An important caveat is that the list of identified putatively damaging variants in ALS genes likely contains a proportion of false positives, because our filtering also identified variants in controls (Additional file 1: Table S9). We identified a similar proportion of ‘oligogenic’ individuals (those that harbour two or more rare variants in ALS genes [from any category; causal, risk, candidate] and/or similar disease genes) in cases and controls (4% vs 3%) (Additional file 1: Table S10). Notably, no individual harboured more than one likely causal variant demonstrating that these results cannot yet provide any evidence for an oligogenic, rare variant basis in ALS.

## Conclusions

It is well recognised that large sample sizes are needed to detect association of rare variants in complex diseases, such as ALS [43]. Despite being the third largest WES study for ALS and the largest such study in Chinese to date, our study remains limited by sample size. We provide novel evidence for association of *NEK1* with ALS in Chinese, reporting variants in these genes not previously found in Europeans. To increase the power for discovery, combining our study with other whole-exome studies (or genome studies) is warranted. To facilitate future meta-analyses, we report per gene counts of all WES variants that pass filtering steps in Chinese (Additional file 1: Tables S5 and S6) and list those variants with ALS-relevant annotation (Additional file 1: Tables S7–S9 and S11),

## Additional files

Additional file 1: Table S1. Detailed information of samples. **Table S2.** Analysis of rare coding variants. **Table S3.** Description and summary of quality control steps of whole-exome sequencing samples. **Table S4.** Exome sequencing coverage in genes included in the WES analyses. **Table S5.** Gene-based SKAT-O association test p-values and per gene counts of WES variants that passed filtering steps. **Table S6.** Gene-based Cochran–Mantel–Haenszel association test results based on WES variant counts in Chinese and Europeans. **Table S7.** Previously reported variants that are likely causal for ALS identified in individuals in our study. **Table S8.** NEK1, SOD1, TBK1 variants identified in SKAT-O and/or ALS specific variant/gene testing. **Table S9.** Not previously reported variants of probable/possible/unknown significance in ALS-related genes identified in at least one individual in our study. **Table S10.** Individuals identified with two or more variants considered relevant for ALS (oligogenic). **Table S11.** Previously reported variants that are unlikely causal (due to high minor allele frequency) identified in individuals in our study. (XLSX 7252 kb)

Additional file 2: Figure S1. Quantile–quantile plots for exome-wide gene-based testing of rare coding variants in the primary analysis of 610 cases and 460 controls. (DOCX 235 kb)

## Abbreviations

ALS: Amyotrophic lateral sclerosis
CHB: Han Chinese in Beijing
ExAC: Exome aggregation consortium
fALS: Familial amyotrophic lateral sclerosis
GWAS: Genome-wide association study
HGMD: Human genome mutation database
HNU: Hunan Normal University
QC: Quality control
sALS: Sporadic ALS
SKAT: Sequence kernel association test
SKAT-O: Sequence kernel association test – optimal
WES: Whole-exome sequencing
WMU: Wenzhou Medical University
