# Supplementary material for: Whole-exome sequencing in amyotrophic lateral sclerosis suggests NEK1 is a risk gene in Chinese
Source: Genome Med. 2017 Nov 17;9:97. doi: 10.1186/s13073-017-0487-0 (PMC5693798; doi:10.1186/s13073-017-0487-0)
Supplement: Supplementary file 2 — Quantile–quantile plots for exome-wide gene-based testing of rare coding variants in the primary analysis of 610 cases and 460 controls. (DOCX 235 kb) [file 13073_2017_487_MOESM2_ESM.docx]

**Whole-Exome Sequencing of ALS Provides Evidence that *NEK1* is a Risk Gene in Chinese**

## Fig. S1. Quantile-quantile (QQ) plots for exome-wide gene-based testing of rare coding variants in the primary analysis of 610 cases and 460 controls.

##
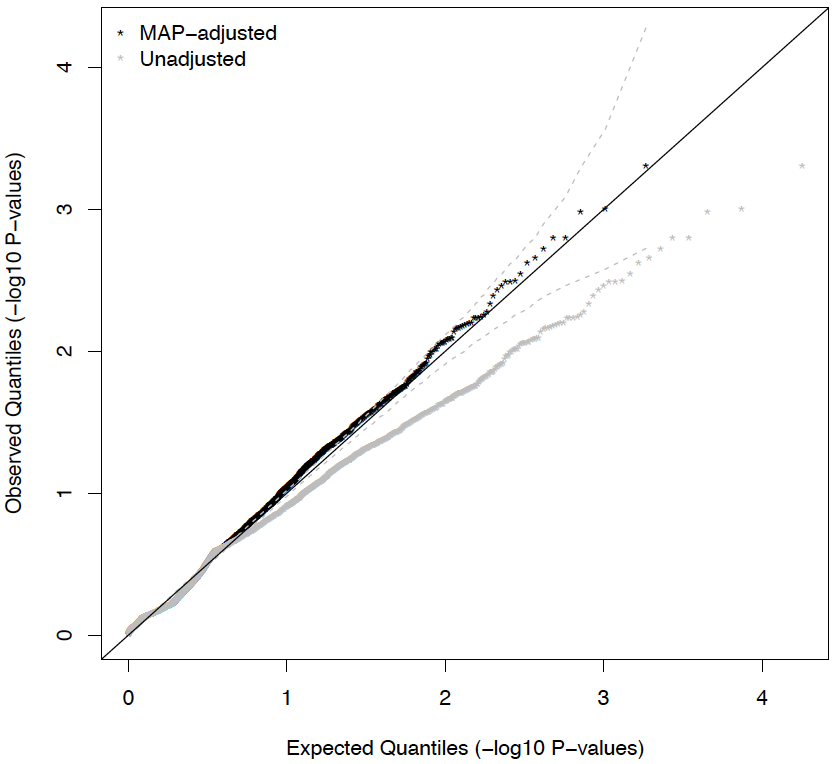
a) b)

**
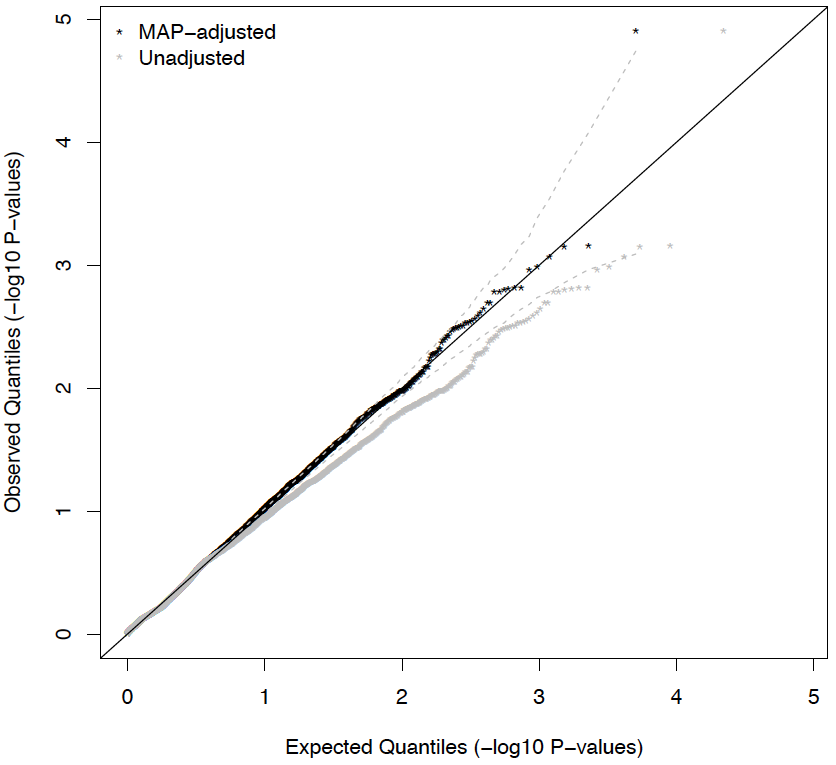
 c)**

**
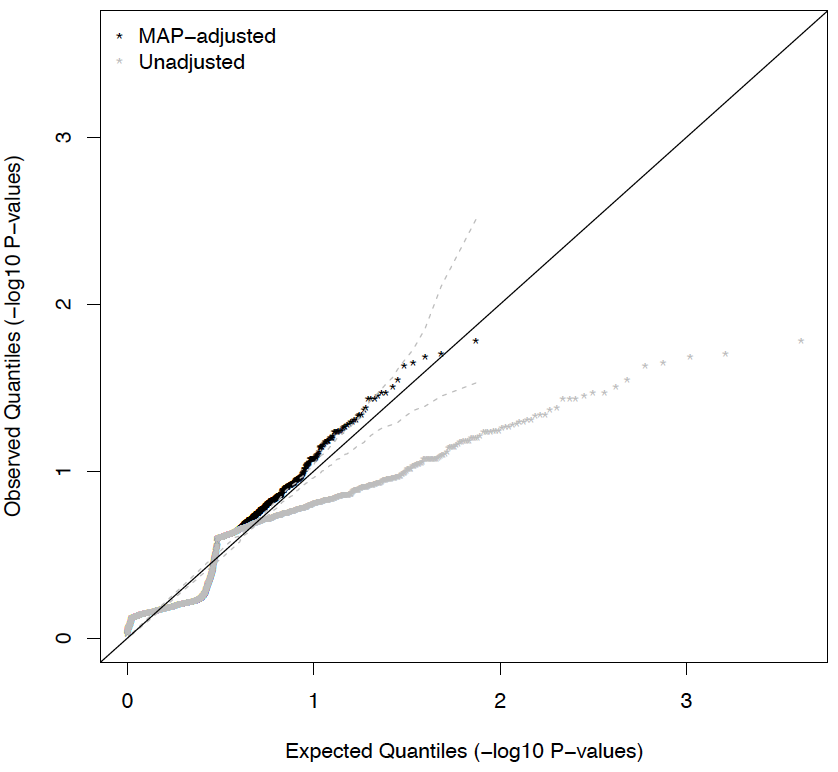
**

a) all nonsynonymous variants, b) nonsynonymous variants excluding those predicted to be benign by PolyPhen-2, c) loss-of-function variants [defined as stop-loss, stop-gain, splicing]), for variants with minor allele frequency <5 x 10 ^-4^. Minimum achievable P-value (MAP)-adjusted results in black and unadjusted results in grey. QQ plots for analysis of Nimblegen version 2 and 3 common capture regions (610 cases and 545 controls) were qualitatively similar. [1-31] [32] [33] [34] [35] [36] [37] [38] [39] [40] [41] [42] [43]

**References for Additional file 1 Table S7**

1. Liu Z-J, Li H-F, Tan G-H, Tao Q-Q, Ni W, Cheng X-W, Xiong Z-Q, Wu Z-Y: **Identify mutation in amyotrophic lateral sclerosis cases using HaloPlex target enrichment system**. *Neurobiology of aging* 2014, **35**(12):2881.e2811-2881.e2815.

2. Puls I, Jonnakuty C, LaMonte BH, Holzbaur ELF, Tokito M, Mann E, Floeter MK, Bidus K, Drayna D, Oh SJ *et al*: **Mutant dynactin in motor neuron disease**. *Nature genetics* 2003, **33**(4):455-456.

3. Ahmed S, Sun S, Siglin AE, Polenova T, Williams JC: **Disease-associated mutations in p150(Glued) destabilizes CAP-gly domain**. *Biochemistry* 2010, **49**(25):5083-5085.

4. Kwiatkowski TJ, Bosco DA, Leclerc AL, Tamrazian E, Vanderburg CR, Russ C, Davis A, Gilchrist J, Kasarskis EJ, Munsat T: **Mutations in the FUS/TLS gene on chromosome 16 cause familial amyotrophic lateral sclerosis**. *Science (New York, NY)* 2009, **323**.

5. Hewitt C, Kirby J, Highley J, et al.: **Novel fus/tls mutations and pathology in familial and sporadic amyotrophic lateral sclerosis**. *Archives of Neurology* 2010, **67**(4):455-461.

6. Dhar SK, Zhang J, Gal J, Xu Y, Miao L, Lynn BC, Zhu H, Kasarskis EJ, St. Clair DK: **FUsed in Sarcoma Is a Novel Regulator of Manganese Superoxide Dismutase Gene Transcription**. *Antioxidants & Redox Signaling* 2014, **20**(10):1550-1566.

7. Dormann D, Rodde R, Edbauer D, Bentmann E, Fischer I, Hruscha A, Than ME, Mackenzie IRA, Capell A, Schmid B *et al*: **ALS-associated fused in sarcoma (FUS) mutations disrupt Transportin-mediated nuclear import**. *The EMBO Journal* 2010, **29**(16):2841-2857.

8. Vance C, Rogelj B, Hortobagyi T, Vos KJ, Nishimura AL, Sreedharan J, Hu X, Smith B, Ruddy D, Wright P: **Mutations in FUS, an RNA processing protein, cause familial amyotrophic lateral sclerosis type 6**. *Science (New York, NY)* 2009, **323**.

9. Damme PV, Goris A, Race V, Hersmus N, Dubois B, Bosch LVD, Matthijs G, Robberecht W: **The occurrence of mutations in FUS in a Belgian cohort of patients with familial ALS**. *European journal of neurology* 2010, **17**(5):754-756.

10. Coady TH, Manley JL: **ALS mutations in TLS/FUS disrupt target gene expression**. *Genes & Development* 2015, **29**(16):1696-1706.

11. Mochizuki Y, Isozaki E, Takao M, Hashimoto T, Shibuya M, Arai M, Hosokawa M, Kawata A, Oyanagi K, Mihara B *et al*: **Familial ALS with FUS P525L mutation: two Japanese sisters with multiple systems involvement**. *Journal of the Neurological Sciences* 2012, **323**(1):85-92.

12. Belzil VV, Valdmanis PN, Dion PA, Daoud H, Kabashi E, Noreau A, Gauthier J, for the SDt, Hince P, Desjarlais A *et al*: **Mutations in FUS cause FALS and SALS in French and French Canadian populations**. *Neurology* 2009, **73**(15):1176-1179.

13. Yulug IG, Katsanis N, de Belleroche J, Collinge J, Fisher EMC: **An improved protocol for the analysis of SOD1 gene mutations, and a new mutation in exon 4**. *Human molecular genetics* 1995, **4**(6):1101-1104.

14. Byström R, Andersen PM, Gröbner G, Oliveberg M: **SOD1 Mutations Targeting Surface Hydrogen Bonds Promote Amyotrophic Lateral Sclerosis without Reducing Apo-state Stability**. *Journal of Biological Chemistry* 2010, **285**(25):19544-19552.

15. Ayers J, Lelie H, Workman A, Prudencio M, Brown H, Fromholt S, Valentine J, Whitelegge J, Borchelt D: **Distinctive features of the D101N and D101G variants of superoxide dismutase 1; two mutations that produce rapidly progressing motor neuron disease**. *Journal of neurochemistry* 2014, **128**(2):305-314.

16. Ogasawara M, Matsubara Y, Narisawa K, Aoki M, Nakamura S, Itoyama Y, Abe K: **Mild ALS in Japan associated with novel SOD mutation**. *Nature genetics* 1993, **5**(4):323-324.

17. Das A, Plotkin SS: **Mechanical Probes of SOD1 Predict Systematic Trends in Metal and Dimer Affinity of ALS-Associated Mutants**. *Journal of Molecular Biology* 2013, **425**(5):850-874.

18. Ohi T, Nabeshima K, Kato S, Yazawa S, Takechi S: **Familial amyotrophic lateral sclerosis with His46Arg mutation in Cu/Zn superoxide dismutase presenting characteristic clinical features and Lewy body-like hyaline inclusions**. *Journal of the Neurological Sciences* 2004, **225**(1):19-25.

19. Millecamps S, Salachas F, Cazeneuve C, Gordon P, Bricka B, Camuzat A, Guillot-Noël L, Russaouen O, Bruneteau G, Pradat P-F *et al*: **SOD, ANG, VAPB, TARDBP and FUS mutations in familial amyotrophic lateral sclerosis: genotype–phenotype correlations**. *Journal of medical genetics* 2010, **47**(8):554.

20. Xiong HY, Alipanahi B, Lee LJ, Bretschneider H, Merico D, Yuen RKC, Hua Y, Gueroussov S, Najafabadi HS, Hughes TR *et al*: **The human splicing code reveals new insights into the genetic determinants of disease**. *Science (New York, NY)* 2015, **347**(6218):1254806-1254806.

21. Shaw CE, Enayat ZE, Chioza BA, Al-Chalabi A, Radunovic A, Powell JF, Leigh PN: **Mutations in all five exons of SOD-1 may cause ALS**. *Annals of neurology* 1998, **43**(3):390-394.

22. Ebben MR, Shahbazi M, Lange DJ, Krieger AC: **REM behavior disorder associated with familial amyotrophic lateral sclerosis**. *Amyotrophic Lateral Sclerosis* 2012, **13**(5):473-474.

23. Fujisawa T, Homma K, Yamaguchi N, Kadowaki H, Tsuburaya N, Naguro I, Matsuzawa A, Takeda K, Takahashi Y, Goto J *et al*: **A novel monoclonal antibody reveals a conformational alteration shared by amyotrophic lateral sclerosis-linked SOD1 mutants**. *Annals of neurology* 2012, **72**(5):739-749.

24. Eisen A, Mezei MM, Stewart HG, Fabros M, Gibson G, Andersen PM: **SOD1 gene mutations in ALS patients from British Columbia, Canada: clinical features, neurophysiology and ethical issues in management**. *Amyotrophic lateral sclerosis : official publication of the World Federation of Neurology Research Group on Motor Neuron Diseases* 2008, **9**(2):108-119.

25. Pramatarova A, Figlewicz DA, Krizus A, Han FY, Ceballos-Picot I, Nicole A, Dib M, Meininger V, Brown RH, Rouleau GA: **Identification of new mutations in the Cu/Zn superoxide dismutase gene of patients with familial amyotrophic lateral sclerosis**. *American Journal of Human Genetics* 1995, **56**(3):592-596.

26. Kwon M-J, Baek W, Ki C-S, Kim HY, Koh S-H, Kim J-W, Kim SH: **Screening of the SOD1, FUS, TARDBP, ANG, and OPTN mutations in Korean patients with familial and sporadic ALS**. *Neurobiology of aging* 2012, **33**(5):1017.e1017-1017.e1023.

27. Akimoto C, Morita M, Atsuta N, Sobue G, Nakano I: **High-Resolution Melting (HRM) Analysis of the Cu/Zn Superoxide Dismutase (SOD1) Gene in Japanese Sporadic Amyotrophic Lateral Sclerosis (SALS) Patients**. *Neurology Research International* 2011, **2011**:165415.

28. Furukawa Y, Kaneko K, Yamanaka K, Nukina N: **Mutation-dependent Polymorphism of Cu,Zn-Superoxide Dismutase Aggregates in the Familial Form of Amyotrophic Lateral Sclerosis**. *Journal of Biological Chemistry* 2010, **285**(29):22221-22231.

29. Shimizu T, Kawata A, Kato S, Hayashi M, Takamoto K, Hayashi H, Hirai S, Yamaguchi S, Komori T, Oda M: **Autonomic failure in ALS with a novel SOD1 gene mutation**. *Neurology* 2000, **54**(7):1534-1537.

30. Rosen DR, Siddique T, Patterson D, Figlewicz DA, Sapp P, Hentati A, Donaldson D, Goto J, O'Regan JP, Deng HX *et al*: **Mutations in Cu/Zn superoxide dismutase gene are associated with familial amyotrophic lateral sclerosis**. *Nature* 1993, **362**(6415):59-62.

31. Brown JA, Min J, Staropoli JF, Collin E, Bi S, Feng X, Barone R, Cao Y, O'Malley L, Xin W *et al*: **SOD1, ANG, TARDBP and FUS mutations in amyotrophic lateral sclerosis: a United States clinical testing lab experience**. *Amyotrophic lateral sclerosis : official publication of the World Federation of Neurology Research Group on Motor Neuron Diseases* 2012, **13**(2):217-222.

32. Kirby J, Goodall EF, Smith W, Highley JR, Masanzu R, Hartley JA, Hibberd R, Hollinger HC, Wharton SB, Morrison KE *et al*: **Broad clinical phenotypes associated with TAR-DNA binding protein (TARDBP) mutations in amyotrophic lateral sclerosis**. *Neurogenetics* 2010, **11**(2):217-225.

33. Budini M, Romano V, Avendaño-Vázquez SE, Bembich S, Buratti E, Baralle FE: **Role of selected mutations in the Q/N rich region of TDP-43 in EGFP-12xQ/N-induced aggregate formation**. *Brain research* 2012, **1462**:139-150.

34. Corrado L, Ratti A, Gellera C, Buratti E, Castellotti B, Carlomagno Y, Ticozzi N, Mazzini L, Testa L, Taroni F: **High frequency of TARDBP gene mutations in Italian patients with amyotrophic lateral sclerosis**. *Human mutation* 2009, **30**.

35. Piaceri I, Del Mastio M, Tedde A, Bagnoli S, Latorraca S, Massaro F, Paganini M, Corrado A, Sorbi S, Nacmias B: **Clinical heterogeneity in Italian patients with amyotrophic lateral sclerosis**. *Clinical genetics* 2012, **82**(1):83-87.

36. Origone P, Caponnetto C, Bandettini Di Poggio M, Ghiglione E, Bellone E, Ferrandes G, Mancardi GL, Mandich P: **Enlarging clinical spectrum of FALS with TARDBP gene mutations: S393L variant in an Italian family showing phenotypic variability and relevance for genetic counselling**. *Amyotrophic Lateral Sclerosis* 2010, **11**(1-2):223-227.

37. Rutherford NJ, Zhang YJ, Baker M, Gass JM, Finch NA, Xu YF, Stewart H, Kelley BJ, Kuntz K, Crook RJ *et al*: **Novel mutations in TARDBP (TDP-43) in patients with familial amyotrophic lateral sclerosis**. *PLoS genetics* 2008, **4**(9):e1000193.

38. Floris G, Borghero G, Cannas A, Di Stefano F, Murru MR, Corongiu D, Cuccu S, Tranquilli S, Cherchi MV, Serra A *et al*: **Clinical phenotypes and radiological findings in frontotemporal dementia related to TARDBP mutations**. *Journal of Neurology* 2015, **262**(2):375-384.

39. Kabashi E, Valdmanis PN, Dion P, Spiegelman D, McConkey BJ, Vande Velde C, Bouchard JP, Lacomblez L, Pochigaeva K, Salachas F *et al*: **TARDBP mutations in individuals with sporadic and familial amyotrophic lateral sclerosis**. *Nature genetics* 2008, **40**(5):572-574.

40. Voigt A, Herholz D, Fiesel FC, Kaur K, Müller D, Karsten P, Weber SS, Kahle PJ, Marquardt T, Schulz JB: **TDP-43-Mediated Neuron Loss In Vivo Requires RNA-Binding Activity**. *PloS one* 2010, **5**(8):e12247.

41. Van Deerlin VM, Leverenz JB, Bekris LM, Bird TD, Yuan W, Elman LB, Clay D, Wood EM, Chen-Plotkin AS, Martinez-Lage M *et al*: **TARDBP mutations in amyotrophic lateral sclerosis with TDP-43 neuropathology: a genetic and histopathological analysis**. *The Lancet Neurology* 2008, **7**(5):409-416.

42. Alami NH, Smith RB, Carrasco MA, Williams LA, Winborn CS, Han SSW, Kiskinis E, Winborn B, Freibaum BD, Kanagaraj A *et al*: **Axonal transport of TDP-43 mRNA granules in neurons is impaired by ALS-causing mutations**. *Neuron* 2014, **81**(3):536-543.

43. Araki W, Minegishi S, Motoki K, Kume H, Hohjoh H, Araki YM, Tamaoka A: **Disease-Associated Mutations of TDP-43 Promote Turnover of the Protein Through the Proteasomal Pathway**. *Molecular neurobiology* 2014, **50**(3):1049-1058.
